# Supplementary material for: RNA-Seq Analysis of Trans-Differentiated ARPE-19 Cells Transduced by AAV9-AIPL1 Vectors
Source: Int J Mol Sci. 2023 Dec 22;25(1):197. doi: 10.3390/ijms25010197 (PMC10778816; doi:10.3390/ijms25010197)
Supplement: Supplementary file 1 [file ijms-25-00197-s001.zip › ijms-2752803-supplementary.pdf]

International Journal of *Molecular Sciences*

MDPI

Supplementary materials

>ATGGACGCCGCTCTGCTGCTGAATGTGGAAGGCGTGAAAAAGACCATCCTGCACGGTGGAACAGGCGAGCTGCCTAACTTCATCACCGG
CTCCAGAGTGATCTTCCACTTCCGGACCATGAAGTGCGACGAAGAGAGAACCGTGATTGACGATAGCAGACAGGTGGGCCAGCCTATGCA
CATCATCATCGGCAACATGTTTAAGCTGGAGGTCTGGGAAATCCTGCTGACCAGCATGAGAGTGCATGAAGTTGCCGAGTTCTGGTGCGAT
ACAATCCACACAGGAGTGTACCCTATCCTGTCTAGAAGCCTGAGACAGATGGCCCAGGGAAAGGACCCCACAGAATGGCACGTGCATAC
CTGCGGCCTGGCCAATATGTTCGCTTACCACACCCTGGGATACGAGGACCTGGATGAGCTTCAGAAGGAGCCACAGCCTCTGGTCTTTGTG
ATCGAGCTGCTTCAAGTGGACGCCCCTAGCGACTACCAGCGGGAAACCTGGAACCTGAGCAACCACGAAAAGATGAAAGCTGTGCCTGT
GCTGCACGGCGAAGGCAATCGGCTGTTCAAACTGGGCAGATACGAGGAGGCCAGCAGCAAGTACCAGGAGGCCATCATTTGTTTGCGCA
ACCTGCAGACCAAGGAAAAACCTTGGGAGGTGCAGTGGCTGAAGCTGGAGAAGATGATCAACACCCTGATCCTGAACTACTGCCAGTGT
CTGCTGAAGAAAGAGGAGTACTACGAGGTCCTGGAGCACACATCTGACATCCTGAGACACCACCCCGGCATCGTGAAGGCCTATTACGTG
CGGGCCCGGGCCCACGCCGAAGTGTGGAACGAGGCCGAGGCCAAGGCGGACCTCCAGAAAGTGCTGGAGCTGGAGCCGTCCATGCAGA
AGGCGGTGCGCAGGGAGCTGAGGCTGCTGGAGAACCGCATGGCGGAGAAGCAGGAGGAGGAGCGGCTGCGCTGCCGGAACATGCTGAG
CCAGGGTGCCACGCAGCCTCCCGCAGAGCCACCCACAGAGCCACCCGCACAGTCATCCACAGAGCCACCTGCAGAGCCACCCACAGCAC
CATCTGCAGAGCTGTCCGCAGGGCCCCCTGCAGAGCCAGCCACAGAGCCACCCCCGTCCCCAGGGCACTCGCTGCAGCACTAATGA*

Supplementary Figure S1. AIPL1 codon-optimized sequence

Supplementary Table S1. List of marker genes and qPCR primers.

Gene	Forward primer	Reverse primer	Fluorescence Probe
PPIA	5'-ATGCTGGACCCAACACAAAT -3'	5'-TCTTTCACTTTGCCAAACACC -3'	5′-HEX-CATCTGCACTGCCAAGAC -BHQ1-3′
GAPDH	5'- AGCCACATCGCTCAGACAC -3'	5'- GCCCAATACGACCAAATCC -3'	5′- HEX -GGAAGGTGAAGGTCGGAG -BHQ1-3′
TBP	5'-TTGGGTTTTCCAGCTAAGTTCT-3'	5'-CCAGGAAATAACTCTGGCTCA -3'	5′- HEX -AGCTGTGATGTGAAGTTTCC -BHQ1-3′
**Retinal markers**			
RPE65	5'- ACTTCTCCTTTCAACCTCTTCC-3'	5'- TTTTCACCTCTTCCCAGTTCTC-3'	5′- FAM - AATGGGTTTCTGATTGTGGATCTCTGCT-BHQ1-3′
RDH12	5'-GCCTATTGCCACAGCAAGC-3'	5'-GCAGAGCAGGGAGGAGTG-3'	5′- FAM - GTCACCACCTACGCAGTGCACCCAG-BHQ1-3′
**Photoreceptor markers**			
ARR3	5'- GAAACGGGACTTCGTGGACCATG-3'	5'- CGGCCATAGCGAAAGGCAC-3'	5′- FAM - CCAGGACAACACCGTCAATGGGTTCC-BHQ1-3′
CRX	5'- TGTTTGCCAAGACCCAGTAC-3'	5'- ATTTAGCCCTCCGGTTCTTG-3'	5′- FAM - TCAGGCAGATTGATCTTCAGAGCCAC-BHQ1-3′
CRB1	5'- TGTGCCAGGACTTACTCAAC-3'	5'- AGCAAGAGGATCAGTAACAAGG-3'	5′- FAM - CAGCAAAGGCAACATCACAGAGGC-BHQ1-3′
PDE6B	5'- ACTCCGAGATCGTCTTCCCAC-3'	5'- CCACGACGTCTTTGCCATTC-3'	5′- FAM – CTCATTTGCTGACGAGCTCACTGACTAC-BHQ1-3′
TULP1	5'- GCCAATTACCTCATCTCCATCG-3'	5'- ACATTAGTGCTGTACCCACG-3'	5′- FAM- TTCTGCCCGTTGTCAAAGACCGT-BHQ1-3′
GUCY2D	5'- CCTGCTCAACGATCTCTACAC-3'	5'- TGACATGTTGGCGATCTCTG-3'	5′- FAM- AGGCGTCCCCTATTGTCTCCACT-BHQ1-3′
**Neuronal markers**			
OPN3	5'- GCTCTACTCACTGGCGTGG-3'	5'- AGGAGGAATCGTTGGCATC-3'	5′- FAM- CTGGACGTACACGGACTAGGCTGC-BHQ1-3′
TUBB3	5'- GGGCCAAGTTCTGGGAAGTC-3'	5'- CGAGTCGCCCACGTAGTTG	5′- FAM- ATGAGCATGGCATCGACCCCAGC-BHQ1-3′
**Genes of AIPL1-interacting proteins according to STRING**			
AIPL1	5'- GTGGCTGAAGCTGGAGAAG-3'	5'- CTCCAGCTCCAGCACTTTC -3'	WT: 5′-FAM- AGTGCCTGCTGAAGAAGGAG-BHQ1-3′<br>OPT: 5′-FAM- CAACACCCTGATCCTGAAC BHQ1-3′
NUB1	5'- TCAGAGACTGGTCCACATAAAAG-3'	5'- AGCTGACGTGCCTTGTTAAG-3'	5′- FAM- TCCACTGTGATAGTTTCGGATCCCTTGA-BHQ1-3′
AHR	5'- CCAACATCACCTACGCCAG-3'	5'- CCAAACGGTCCAACTCTGTAT-3'	5′- FAM- CGGTGCAGAAAACGTAAAGCCAATCC-BHQ1-3′
LCA5	5'- TCTAATCGTCTGCCAAAGTCC-3'	5'- TCTGGCTTGAAGTCTTCCATG-3'	5′- FAM- TGTACACAGGTCTGCAAAATCACTCTGG-BHQ1-3′
**Transcription factors**			
KLF4	5'- ACCAGGCACTACCGTAAACACA-3'	5'- GGTCCGACCTGGAAAATGCT-3'	5′- FAM- CCAGTGCCAAAAATGCGACCG -BHQ1-3′

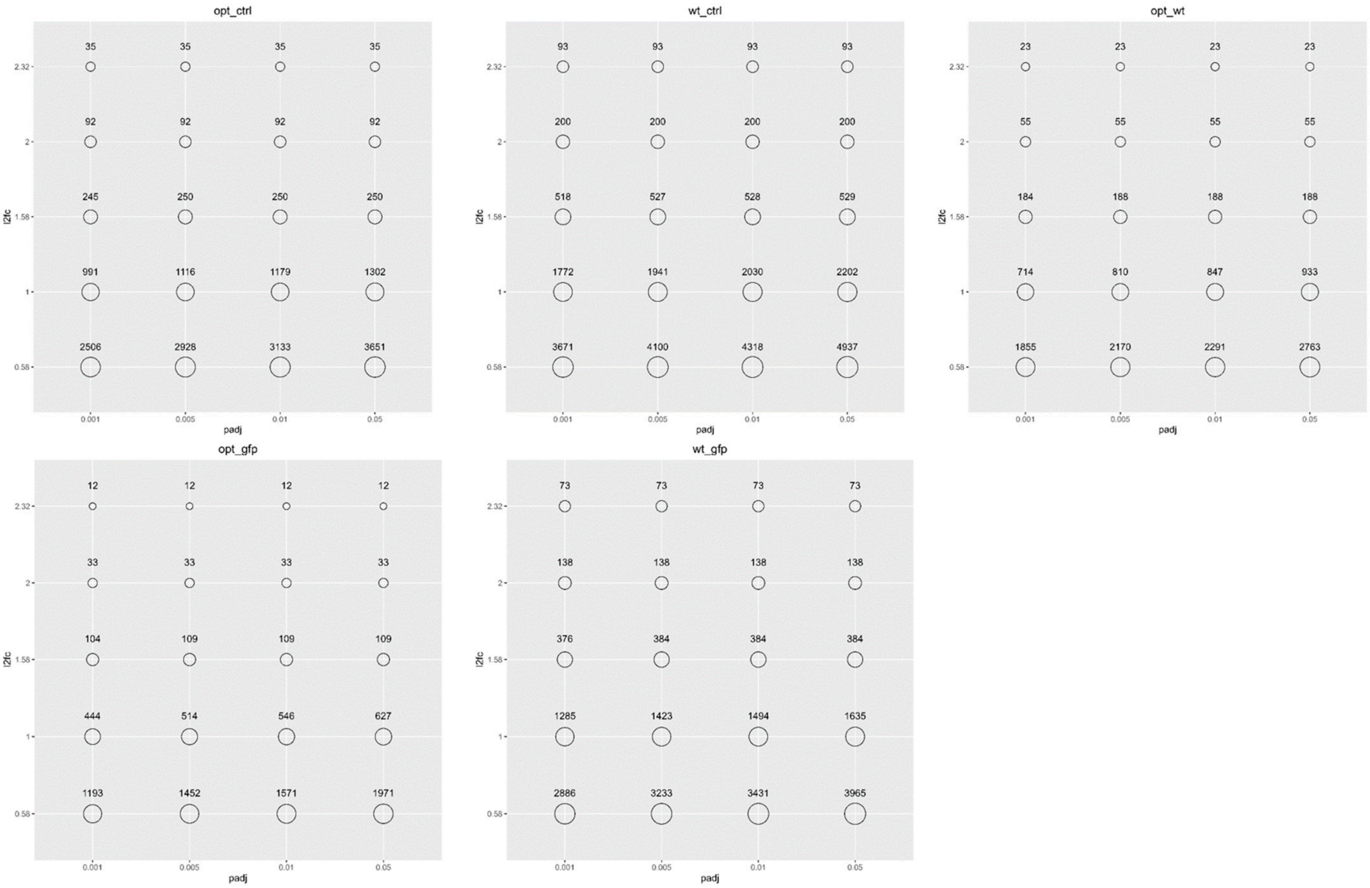

Supplementary Figure S2. Numbers of differentially expressed genes with various thresholds (p-value adjusted and fold change).

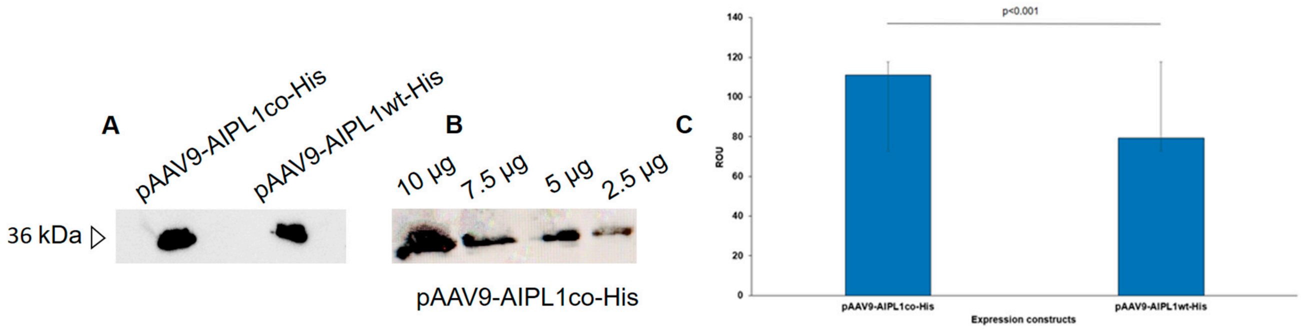

Supplementary Figure S3. Analysis of AIPL1 expression. AIPL1 gene product was detected by western blot using HEK293T cells transfected with pAAV-AIPL1co-His and pAAV-AIPL1wt-His plasmids. AIPL1 protein specific band at 36 kDa was detected in cell lysates with anti-His antibodies (A). Various amount of HEK293T cell lysate was loaded in SDS-PAGE (B). Relative optical density units (RDU) of protein specific bands were calculated and plotted in a graph to visualize the difference between pAAV-AIPL1co-His and pAAV-AIPL1wt-His transfected cells (C). AAV-AIPL1co-His showed 1.39 time increase of overall protein product compared to pAAV-AIPL1wt-His transfected HEK-293Tcells.

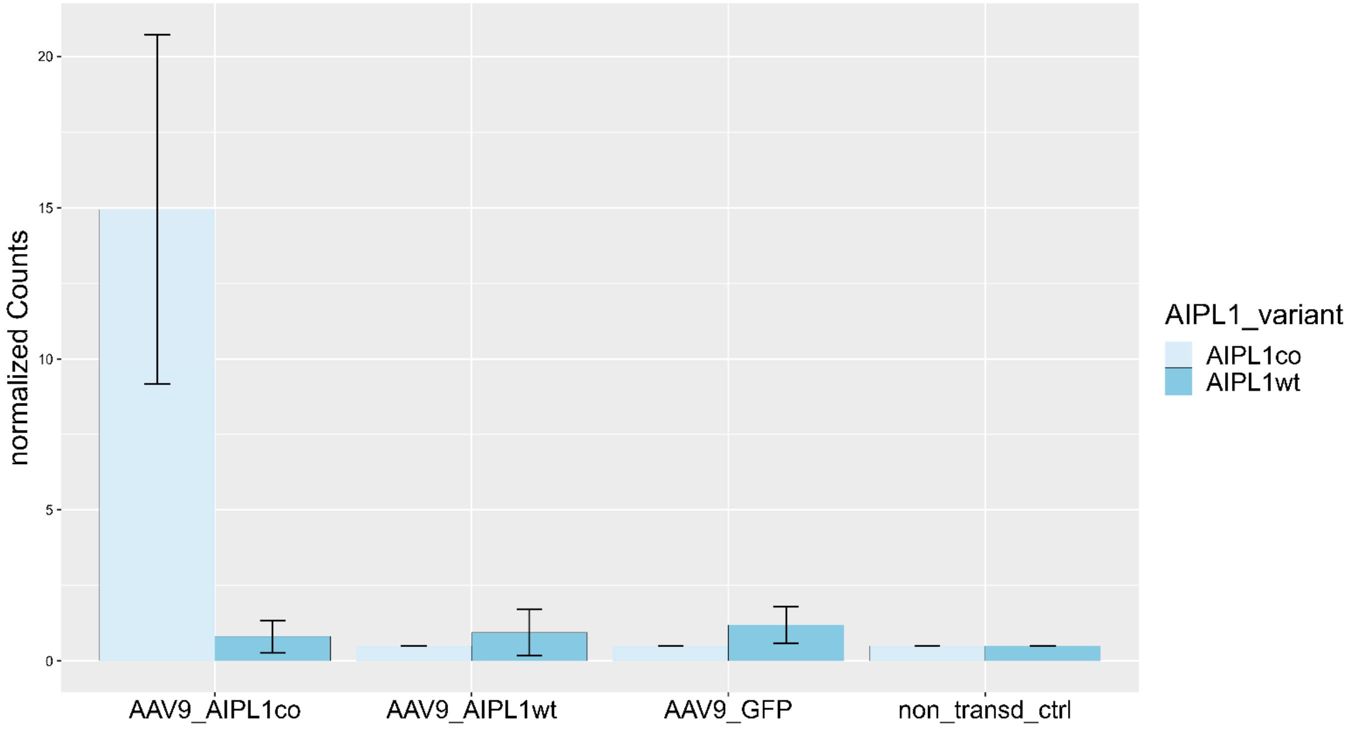

Supplementary Figure S4. RNA-sequencing normalized *AIPL1* gene variants counts. Error bars represent standard deviation between replicates within sample groups.
